# Role of Biosynthesized Ag-NPs Using *Aspergillus niger* (MK503444.1) in Antimicrobial, Anti-Cancer and Anti-Angiogenic Activities

**DOI:** 10.3389/fphar.2021.812474

**Published:** 2022-01-25

**Authors:** Akbar Pasha, Divya Vishambhar Kumbhakar, Siva Sankar Sana, Doneti Ravinder, B. Vijaya Lakshmi, Suresh K. Kalangi, Smita C. Pawar

**Affiliations:** ^1^ Department of Genetics & Biotechnology, Osmania University, Hyderabad, India; ^2^ School of Chemical Engineering and Technology, North University of China, Taiyuan, China; ^3^ Institute of Genetics and Hospital for Genetic Diseases, Osmania University, Hyderabad, India; ^4^ Amity Stem Cell Institute, Amity Medical School, Amity University Haryana, Gurgaon, India

**Keywords:** *Aspergillus niger*, Ag-NPs, Antimicrobial activity, wound healing, invasion, Apoptosis, Angiogenesis

## Abstract

Green synthesis of nanoparticles is regarded as a safe and non-toxic process over conventional synthesis. Owing to the medicinal value of biologically derived biomolecules and utilizing them in synergy with nanoscience to offer more accurate therapeutic options to various diseases is an emerging field. One such study we present here with highlights of the synthesis and efficacy of biogenic silver nanoparticles produced from the extract of *Aspergillus niger SAP2211* (accession number: *MK503444.1*) as an antimicrobial, anti-cancerous and anti-angiogenic agent. The synthesized Ag-NPs were characterized following UV–vis, FTIR, XRD, SEM and TEM, and were found to possess bactericidal activity against the selected pathogenic microbes, such as *Staphylococcus aureus, Escherichia coli,* and *Salmonella typhi*. Further, we evaluated cytotoxicity effect of this biogenic Ag-NPs using MMT assay on normal cardio myoblast (H9C2) and cancerous human cervical carcinoma (HeLa) cells. Doxorubicin used as positive control. This Ag-NPs have shown trivial cytotoxicity at the IC_50_ concentration on normal cells (IC_50_ = 47.17 µg/ml) over the cancer cells (IC_50_ = 8.609 µg/ml) with nearly 7 fold difference, indicating it as a selective anti-cancerous agent in contrast to standard drug doxorubicin (IC_50_ = 6.338 µg/ml). Further *in-vitro* assessment of wound healing capability by scratch wound healing assay, invasion by transwell matrigel invasion assay, and apoptosis via DAPI and annexin V-FITC assays were studied in HeLa cells. Synthesized biogenic Ag-NPs have shown to be anti-angiogenic in nature, which was established by *in-vivo* chick chorioallantois membrane assay. Overall, *in vitro* studies revealed that biogenic Ag-NPs positively inhibited migration, invasion, and induced apoptosis, and *in-vivo* CAM assay revealed that intercapillary network was reduced and the angiogenesis was inhibited.

## 1 Introduction

Cancer is a life-threatening disease that is responsible for the majority of fatalities worldwide (Gao et al., 2013). Conventional therapeutic approaches applied for cancer treatment have shown limited long-term survivability of the patients and side effects. As a result, one of the most ardent aim is the creation of robust and effective anti-cancer medications. The biocompatible metal nanoparticles have been extensively employed for medical applications in humans considering the dose implementation of low toxicity. Metal-based silver nanoparticles (Ag-NPs) have sparked interest due to their unique physicochemical properties such as chemical stability and electrical conductivity (Sharma et al., 2009) and also biological properties, including anti-bacterial, anti-fungal, anti-inflammatory, anti-viral, anti-angiogenesis, anti-cancer, and anti-platelet properties (Wong and Liu, 2010; Krishnaraj et al., 2012; Monteiro et al., 2012). Ag-NPs exhibited intrinsic cytotoxic activity in tumor cells (Singh and Ramarao, 2012; El-Sonbaty, 2013) including the release of silver ions and induced oxidative stress in fibroblast and glioblastoma cells, HeLa cells, THP-1 monocytes, breast MCF-7, lung A549, and squamous carcinoma SCC-25 cells (Sonoda et al., 1998; Asharani et al., 2009; Foldbjerg et al., 2009; Abrahamse et al., 2014; Dziedzic et al., 2016; Dayem et al., 2017; El-Hussein and Hamblin, 2017). As a result, cellular pathways become dysregulated, resulting in increased cellular damage and apoptosis (Gomes et al., 2021) thus providing an insight toward the use of nanomedicines. Similarly, silver has been utilized as an antibacterial agent in variety of methods, either alone or in conjunction with other technologies, since decades (Silva et al., 2017). Ag-NPs and its ions are documented to be an efficient antimicrobial agent against a wide range of gram-positive and gram-negative pathogenic bacteria, thereby plummeting the problem of multi-drug resistance (Yoon et al., 2007; Cavassin et al., 2015; Salem et al., 2015; Gahlawat et al., 2016), because of their size similarity, Ag-NPs can interact with and pass through the cell wall and membrane, directly impacting intracellular components.

In order to improve biological applications of Ag-NPs as therapeutic agents, the advancement of environmentally sustainable technology in material synthesis is of significant importance. To overcome their potential hazard and toxic effect, green synthesis have shown exceptional recognition and is preferred over chemical and physical methods because it is cost effective, less toxic, eco-friendly, requires less energy, gives high productivity and significantly biocompatible with high reduction potential (Bhattacharya and Gupta, 2005; Mohanpuria et al., 2008; Prasad, 2014; Thuesombat et al., 2014; Aziz et al., 2015; Aziz et al., 2019; Sana et al., 2020). These biogenic nanoparticles are reported to be synthesized from bacteria (Saifuddin et al., 2009), actinomycetes (Ahmad et al., 2003), plants (Dinesh et al., 2015), sugar (Darroudi et al., 2011), biodegradable polymers-chitosan (Venkatesham et al., 2012), which act as both reducing and stabilizing agents (Durán et al., 2011). For biogenic Ag-NPs synthesis, fungus is documented as an ideal agent over plants and bacteria offering high tolerance towards metals with high wall-binding capacity and intracellular metal uptake and accumulation capability (Dias et al., 2002; Musarrat et al., 2010). The OSMAC approach is used to identify a significant number of undiscovered natural compounds carried by marine fungus (Romano et al., 2018). Myco-synthesis of silver nanoparticles leads to rapid reduction and intracellular accumulation of Ag-NPs in the form of ions (Mukherjee et al., 2001; Bhainsa and D’Souza, 2006; Jha et al., 2009) using acidophilic fungi such as *Verticillium sp, Fusarium oxysporum*, *Penicillium fungi*, *Trichoderma reesei*, and *Aspergillus fumigates*. Ag^+^ ions are trapped at the cell surface after electrostatically interacting with negatively charged carboxylate groups in the mycelial cell wall (Mukherjee et al., 2001), following that, the trapped ions are further reduced by fungal enzymes reductases (Jain et al., 2011; Dhillon et al., 2012). The fungi release extracellular proteins providing stability to NPs (Balaji et al., 2009; Du et al., 2015; Bethu et al., 2016) in order to prevent its aggregation and standardize different characteristics of nanoparticles such as size, charge, and surface morphology. This unusual dimensional feature along with species specific biomolecules provided from fungus makes Ag-NPs to inhibit bacterial replication and have become a method for combating infectious diseases (Murthy, 2007) and promote wound healing (Barillo and Marx, 2014). The nano-silver sythesized using *Aspergillus sydowii,* are documented to show excellent antifungal properties against pathogenic fungi as well as antiproliferative activity against HeLa cells and MCF-7 cells (Wang et al., 2021). Hu et al. (2019) used an endophytic fungus (*Talaromyces purpureogenus*) for Ag-NPs synthesis and found that it exhibited antibacterial, anticancer, and wound healing properties. These Ag-NPs are prospective drug leads due to the positive outcomes of prior research investigations as well as their low cost. It becomes indispensable to design a biocompatible and prolific nanoparticle that can specifically target HeLa carcinoma cells for therapeutic efficacy with reduced side effects on normal cells.

The current research focuses on the production of silver nanoparticles from *Aspergillus niger SAP2211* (a new strain) isolated from a marine sponge, as well as their subsequent characterization to determine standard nano-quality. The synthesized Ag-NPs was tested for antibacterial activity against pathogenic bacteria such as, *Staphylococcus aureus, Escherichia coli,* and *Salmonella typhi*. The anti-cancerous properties were examined *in vitro* in HeLa carcinoma cells and assessed cell viability by MTT assay, apoptosis by DAPI and annexin V FITC/PI, cell migration by wound healing scratch assay, and invasiveness assessed by transwell matrigel invasion assay. The anti-angiogenic potentiality were assessed *in vivo* utilizing the chorioallantoic membrane (CAM) model with commercial doxorubicin serving as positive control. The resulting Ag-NPs showed effective antimicrobial, anti-cancerous and anti-angiogenic potential against cervical HeLa cells with no adverse effect on normal cell, providing a platform for the use of this biogenic nano silver particles as potent antimicrobial, anti-cancerous and an anti-angiogenic agents.

## 2 Materials and Methods

### 2.1 Chemicals and Reagents

All the chemicals and reagents used in the investigation are of molecular grade and purchased from HiMEDIA, Takara, and Sigma-Aldrich laboratories.

### 2.2 Preparation of NPs

#### 2.2.1. Isolation of Fungus From Marine Sponges

Marine sponges were collected from intertidal and subtidal regions (1–5 m depth manually by scuba diving) of Marina Beach sea coast Chennai, India. The sponges were air dried for a period of 3–5 days and were crushed into powder.

One gram of crushed sponge was serially diluted in sterilized distilled water to a concentration of 10^−1^–10^–7^. A volume of 0.1 ml of each dilution was inoculated aseptically in 2 ml of Potato Dextrose Agar (PDA) plates and incubated at 37°C for 72 h. The fungal isolates were subculture on PDA plates in order to obtain pure culture. Pure isolates were then maintained in the laboratory at 4°C in a refrigerator for further studies.

#### 2.2.2 Colony Characterization

The colony morphology of fungal isolates was characterized by Lactophenol Cotton Blue Mounting and was observed under Olympus trinocular light microscope (CH*20i*) for its color, shape, chain morphology, hyphae and mycelium structure.

#### 2.2.3 Molecular Analysis of Identified Fungi

**FIGURE 1:**
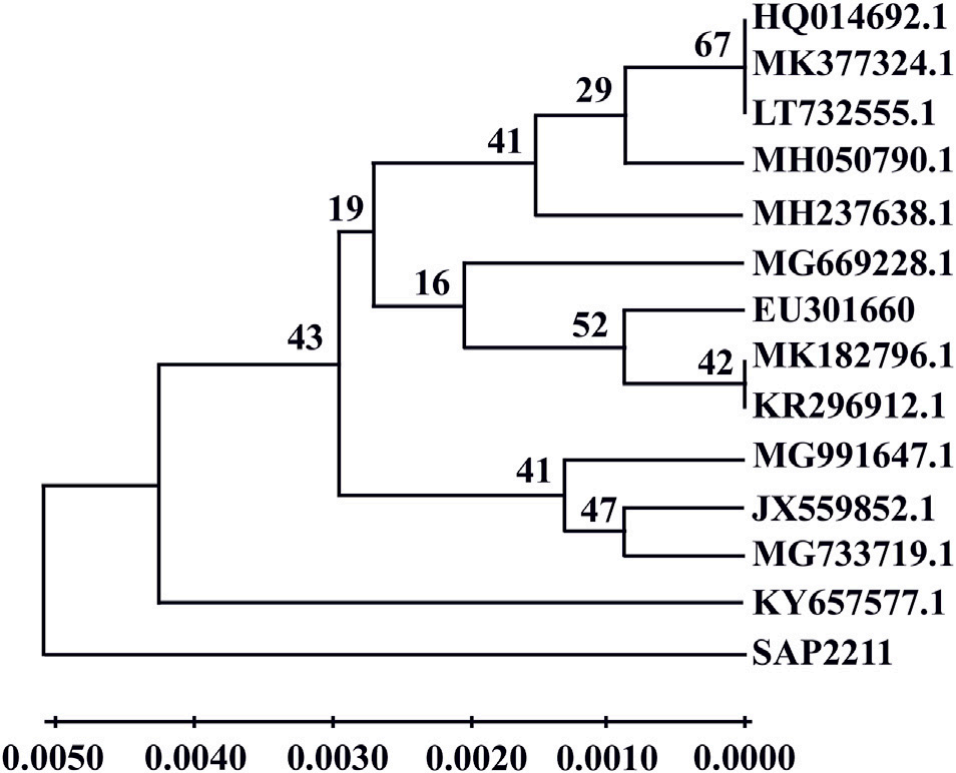
Phylogenetic tree of fungi *Aspergillus niger* sap 2211.

The DNA was isolated from fungal culture using the QIAamp^R^ DNA mini kit (Qiagen). Both quantity and quality of DNA were analyzed in 1% Agarose Tris Acetate EDTA gel. Forward primer-ITS1 (5′-TCC​GTA​GGT​GAA​CCT​GCG​G-3′) and reverse primers-ITS4 (5′-TCC​TCC​GCT​TAT​TGA​TAT​GC-3′) (Murthy, 2007) were used to amplify 18s rDNA by PCR. The reaction mixture (20 µl) set-up consists of 10 µl of master mix (Takara #RR310), 1 µl (10 pmol) each of both forward and reverse primers, 1 µl of template DNA and 7 µl of ddH_2_O. The PCR product was then loaded onto 1.0% agarose gel for electrophoresis and visualized under Gel doc system (Biorad). The amplicon 18s rDNA gene was sequenced using an automated ABI–DNA sequencer (Applied Biosystems 3500) at Centre for Plant Molecular Biology, Osmania University. BLAST was carried out with the NCBI Genbank database using the fungal 18s rDNA ITS gene sequence and deposited in Genbank/EMBL. Sequences were identified and matched based on maximum identity values using the multiple alignment tool Clustal W. MEGA10 was used to generate the phylogenetic tree. (Figure 1).

### 2.3 Biosynthesis of Silver Nanoparticles

Ag-NPs was synthesized following the methodology adopted by Gudikandula et al. (2017). *Aspergillus niger* was cultured in Potato Dextrose Broth (PDB) in a flask then incubated at 25°C at pH 6.0 for 3 days in a rotary orbital shaker at a speed of 200 rpm. Following incubation, the biomass was separated using Whatmann No.1 filter paper and was aseptically washed with sterile distilled water to remove the remaining medium components. Biomass (20 g) was mixed with 200 ml of distilled water in a 500 ml Erlenmeyer flask and incubated (25°C, 3 days) in a rotary shaker. The cell suspension after incubation was filtered with Whatmann No.1 filter paper and collected for nanoparticles synthesis. Equal quantities (1:1 ratio) of both filtered supernatant and AgNO_3_ (1 mM) were mixed in 250 ml Erlenmeyer flask and kept in a shaker (200 rpm, 25°C) and were confirmed by change in its color.

### 2.4 Characterization of Biosynthesized Ag-NPs

The prepared Ag-NPs were characterized to determine their size, morphology and crystallinity by using UV-visible spectroscopy (Shimadzu UV-1800), Fourier transform infra-red spectrophotometer (Jasco FT/IR-6300), X-ray diffractometer (Shimadzu XRD-7000), Scanning electron microscope (JEOL Japan; JFC-1600) and Transmission electron microscope (JEOL JEM-2100HR, resolution 0.14–0.23 nm).

The reduced silver ions were analyzed in the range of 300–600 nm by UV-vis. The size and morphology of the synthesized nanoparticles was determined by Scanning electron microscopy. The sample was filtered through Millipore filters of 0.2 µ to remove any contaminants. Later loaded onto a stub and coated with platinum and were intended mainly to prepare specimens for SEM observation. The size and shape of Ag-NPs were determined by TEM for which the Ag-NPs sample was diluted upto 100 times and were dropped dried on a carbon-coated Cu grid (Applied Biosystems, India) for analysis. The size distributions of the prepared Ag-NPs on the acquired TEM images were assessed using Originpro.

XRD patterns of dried Ag-NPs was analyzed by Philips X-Ray diffractometer (Shimadzu XRD-7000, X-Ray Generator operated at a voltage of 40 kV and a current of 30 mA) using Cu-K_α_ radiation with λ = 1.5406 Å and 2θ (Bragg angle) ranging between 10° and 80° in steps of 0.02**°** with sampling time of 0.60 s per step. Debye Scherrer equation was employed to calculate the crystallite size (D_nkl_ = kλ/β cosθ; where λ = wavelength of CuK_a_ radiation, β = full width of half maximum intensity, θ = diffraction angle in radian and k = shape factor constant).

The pellet was made from dried Ag-NPs with potassium bromide (IR grade) in 1:100 ratio for FTIR analysis. Diffused reflectance was recorded in the range of 4,000–400 cm^−1^ in infrared spectrum.

### 2.5 Bacterial Strains

The bacterial-strains *Staphylococcus aureus* (MTCC 96), *Escherichia coli* (MTCC 443) and *Salmonella typhi* (MTCC 98) were procured from Department of Microbiology, Osmania University and maintained in Luria-Bertani (LB) broth at 37°C for 24 h in orbital shaker incubator.

#### 2.5.1 Antibacterial Activity by Agar Well Diffusion Method

The antibacterial activity of silver nanoparticles was examined using agar well diffusion technique. The broth (0.1 ml) of each strain was uniformly plated on Nutrient agar medium using sterile cotton swabs – HiMEDIA. A well (diameter: 6–8 mm) was punched aseptically using a sterile cork borer on nutrient agar plate and approximately 10 μl volume of the synthesized Ag-NPs (Stock concentration of 1 mg/ml and 500 µg/ml) was injected into the well followed by incubation. This test was carried out in triplicate. As a control, conventional antibiotics (tetracycline, ciprofloxacin) were employed, and the zone of inhibition was determined.

### 2.6 Cell Culture

Myocardial (H9C2) and human cervical carcinoma (HeLa) cell lines were procured from National Centre for Cell Science (NCCS), Pune and were maintained in Dulbecco Modified Eagle’s Medium (DMEM) supplemented with 10% Fetal Bovine Serum (FBS) and 1% penicillin and streptomycin antibiotics. The cells were incubated at 37**°**C in a 5% CO_2_ incubator for 24 h. The cultured cells were passaged using trypsin–EDTA (0.25%) followed by centrifugation (2,000 rpm for 3 min) and resuspended in 1 ml DMEM media. About 100 µl of both cell lines (7500 cells) were added to each well of 96 welled plate and incubated overnight (37**°**C in 5% CO_2_ incubator).

#### 2.6.1 Treatment With Ag-NPs and Doxorubicin

Stock solution of the synthesized Ag-NPs (1 mg/ml) and doxorubicin (1 mg/ml) was filtered using 0.45 µ syringe filter and diluted with culture media to obtain 1:1 to 1:64 dilution. The defined concentration of Ag-NPs testing agent and doxorubicin (positive control) were added in respective wells and incubated at 37**°**C in 5% CO_2_ incubator for 21 h. Untreated cells in DMEM medium were taken as negative control.

#### 2.6.2 Cytotoxicity Assay

Cell viability due to biogenic Ag-NPs and doxorubicin against cultured HeLa cell lines was performed by MTT (3-(4, 5-dimethyl thiazol-2yl)-2, 5-diphenyl tetrazolium bromide) assay and effect of same doses of Ag-NPs were tested for its toxicity on normal H9C2 cells. The degradation of cellular mitochondria has been evaluated by an estimation of blue formazan crystals produced by the reduction of MTT by means of mitochondrial succinic dehydrogenase secreted by viable cells. MTT (20 µl/well of 5 mg/ml in PBS) was added to overnight incubated 96 welled plate followed by incubation for 3–4 h. Mitochondrial dehydrogenase reduces the yellowish water-soluble MTT to water-insoluble formazan crystals that was solubilized by adding 100 µl of DMSO in each well of the plate followed by incubation of 30 min in a shaker incubator. The quantity of formazan which is directly proportional to the number of viable cells, was measured at 590 nm using a spectrophotometer (Bio-Rad). IC_50_ was calculated using Graph-pad Prism software.

#### 2.6.3 Apoptotic Analysis by DAPI (4,6-Diamidino-2-phenylindole Dihydrochloride)

The apparently induced apoptosis by the biogenic Ag-NPs was assessed by monitoring the nuclear cell morphology exposed to various concentrations of Ag-NPs using DAPI staining method. The HeLa cells (2×10^5^ cells/well) were cultured in 6-well plates, incubated at 37°C for overnight. Considering IC_50_ of Ag-NPs assessed by MTT assay, the applied doses of Ag-NPs treatments (5, 10, and 15 µg/ml) for 24 h were compared to the untreated negative controls cells and 10 µg/ml doxorubicin treated positive controls cells. All the cells were fixed with 3.8% paraformaldehyde and stained with DAPI (0.5 µg/ml in PBS) for 15 min at 37°C in dark. The cells were washed twice with PBS. The images were analyzed and captured using an inverted fluorescent microscope (EVOS™ M5000, USA) under 20X magnification.

#### 2.6.4 Scratch Wound Healing Assay

Scratch wound healing assay is convenient and widely performed to determine cell migration capabilities of individual cells, it measures the expansion of individual cell number on edge surface of the scratch (Liang et al., 2007; Muhammad et al., 2013). In the study, HeLa cells were plated in a 6 welled plate (2×10^5^ cells/well) and cultured at 37°C, 5% CO_2_ for 24 h. Untreated cells were considered as negative control and the wells containing 80% confluence (approximately) were taken for the scratch test. An even scratch on cell monolayer was made with a sterile p10 tip micropipette and care was taken to avoid any possible variation in scratch width in treated and control cells, and the floating cells were washed with PBS to clean the edges. The cells were incubated with DMEM complete medium and treated with 5, 10 and 15 μg/ml of Ag-NPs and 10 µg/ml of doxorubicin. Immediately after the procedure i.e. at 0 h the region of initial migration was measured and subsequent measurements were taken after 24 and 40 h of incubations at 37°C, under inverted fluorescence microscope (EVOS™ M5000, USA), and analyzed with ImageJ software (version 1.50i, National Institute of Health, Bethesda, MD, USA) and statistically analyzed. The percentage of relative migration of NP-treated and untreated cells was determined based on the following equation:

$$\mathrm{Relative migration}=\frac{\mathrm{Area of the wound at}\;0\;h-\mathrm{Area of the wound after}\;24/40\;h}{\mathrm{Area of the wound at}\;0\;h}\times 100$$

#### 2.6.5 Transwell Matrigel Invasion Assay

The invasion of Ag-NPs treated HeLa and control (untreated HeLa) cells was performed using transwell chambers (8 μm, genetix Biotech Asia Pvt. Ltd., Gyeonggi-do, Korea). The chamber was pre-coated with Matrigel (BD Biosciences, CA, USA), and later HeLa cells were seeded into the upper chamber with serum free media at a density of 2 × 10^5^ cells per well, and the lower chamber was filled with DMEM medium (700 μl) supplemented with 12% FBS. The cells were treated with 10 and 15 μg/ml of Ag-NPs for 18 h at 37°C and after incubation; the cells were washed twice with PBS. The cells were then fixed with 3.7% formaldehyde for 2 min at room temperature followed by washing with PBS. The cells were permeabilized with 100% methanol for 20 min at room temperature and washed twice with PBS and were stained with crystal violet for 15 min at room temperature followed by washing twice with PBS. Remaining non-invasive cells from both treated and control were removed using cotton swabs. The inhibition of cell invasion ability was assessed by counting the cells and the images were captured at 40X under an inverted microscope (EVOS™ M5000, CA, USA).

#### 2.6.6 Apoptosis Assay by Annexin V FITC/PI

Apoptosis induced by the Ag-NPs was assessed using an Annexin V-FITC apoptosis detection kit (ApoAlert ™ Annexin V-FITC, Takara, CA, USA). HeLa cells (2.0×10^6^ cells/dish) were incubated at 37°C for 24 h, and further treated with various doses of Ag-NPs (5 and 10 μg/ml) then re-incubated for another 24 h. The control cells were not treated with NPs. The cells were trypsinized and the floating cells were pelleted by centrifugation at 2,500 rpm for 2 min. The obtained pellet was washed with ice-cold PBS (1X) and resuspended in 100 μl binding buffer (1X). The cells were stained with both Annexin V-FITC (5 μl) and propidium iodide (10 μl) in dark for 15 min at room temperature and were diluted with 400 μl of binding buffer. Apoptotic and necrotic cells were evaluated by flow cytometer (Becton-Dickinson Immunocytometry System, Sunnyvale, CA, USA) and data analysis was performed using FACs Cell Quest Pro Software.

#### 2.6.7 Chick Chorioallantois Membrane Assay

The chicken egg CAM experiment was utilized to assess the effectiveness of anticancer medication delivery utilizing the synthesized biogenic Ag-NPs. For the purpose, HeLa cells were inoculated onto the CAM membrane of fertilized eggs, resulting in the fast development of blood capillaries, which is a hallmark of tumors. Eight days fertilized eggs were purchased from Venkateshwara Hatcheries Pvt Ltd., Hyderabad, Telangana, India and incubated at 37°C under 55–60% humidity for 3 days. The eggs were grouped as follows: control group (treated with PBS), eggs treated with HeLa cells taken as positive control, eggs treated with 5, 10, and 15 µg/ml concentrations of Ag-NPs but with no HeLa cells, eggs treated with HeLa cells along with 10 µg/ml doxorubicin and eggs treated with both the HeLa cells as well as all the above doses of Ag-NPs.

The surface of the eggs was cleaned with disinfectant prior to the treatment, egg candler was used to monitor the blood vessels and, a small hole was made and opened on snub side using dissection needle in the biosafety hood. On 11th day of incubation, a sterile 33G needle was used to inject 100 µl of individual doses of biogenic Ag-NPs (5, 10, and 15 µg/ml) to both HeLa cell treated CAM and normal CAM surface, and with doxorubicin (10 µg/ml) as positive control. The hole(s) were covered with sterile wax and incubated at 37°C in a humidified incubator for 72 h, all the experiments were setup in triplicates. The eggs were examined for vascularization on the 14th day of incubation using stereomicroscope (Ziess, Munich, Germany). The number of blood vessels in all the eggs was evaluated in percent values.

### 2.7 Statistical Analysis

The statistical analysis was performed using Graphpad Prism version 6 software, USA. The outcomes of three separate experiments were interpreted as mean ± S.D. and data analysis was performed using both one-way and two-way analysis of variance (ANOVA at *p* < 0.05) followed by Tukey’s test.

## 3 Results

The isolated fungus from the marine sponge was screened and used for Ag-NPs synthesis. Further characterized and evaluated for their antimicrobial, anti-cancerous and anti-angiogenic properties.

### 3.1 Characterization of Fungus

Morphological features, molecular analysis and phylogenetic relationships are considered to be necessary for the identification of fungi. Fungal isolates were distinguished based on size, shape, color, colony morphology and conidial ornamentation observed under microscope via LCB mounting. *Aspergillus sp.* was found to have light yellow-colored conidial heads and stipes, vesicles seriation and metula covering with roughness. The molecular characterization of isolated fungi was performed via PCR-based technique by using ITS1-5.8S-rRNA-ITS4 gene sequences. Sequence of fungal r-DNA ITS regions were compared with NCBI for preliminary identification and, the analyzed sequence and Gene bank sequence were aligned by MEGA BLAST to identify the species of the isolated fungus (Figure 1).

#### 3.1.1 Nucleotide Sequence Accession Number

The fungal rDNA-ITS sequence identified in this study was found to be novel and has been deposited in Genbank/EMBL under accession number MK503444.1.

### 3.2 Synthesis and Characterization of Ag-NPs

#### 3.2.1 Visible Observation of Ag-NPs Synthesis

**FIGURE 2:**
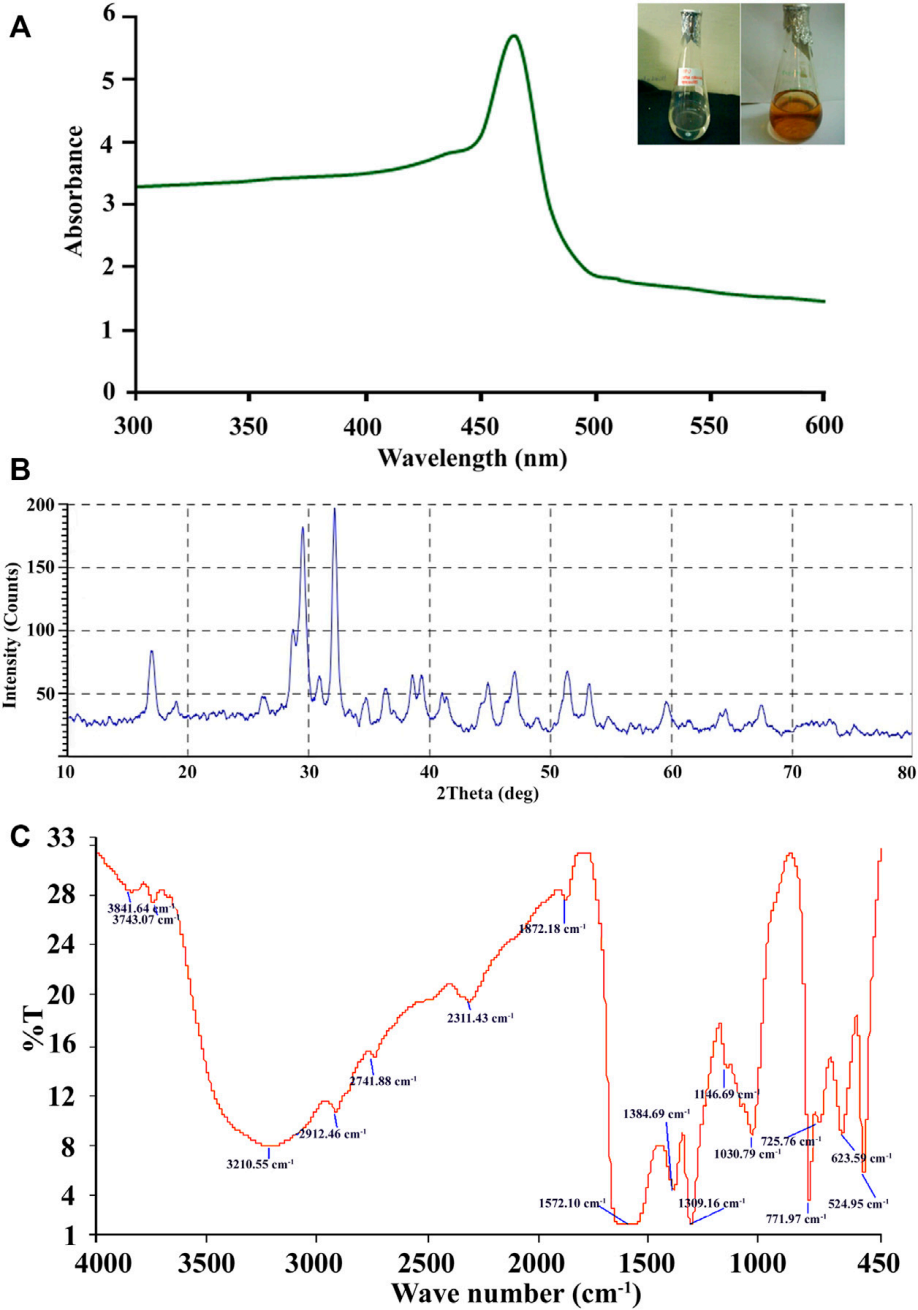
**(A–C)** Biosynthesis of Ag-NPs by *A. niger sap 2211*. **(A)** UV–Visble absorption spectra of extracellularly synthesized Ag-NPs at 460 nm. **(B)** XRD pattern of silver nanoparticles obtained from cell filtrate of *A. niger sap 2211*. **(C)** FTIR spectrum showing associated functional group in Ag-NPs.

A change from colorless to reddish brown color of the synthesized Ag-NPs solution indicates the reduction of aqueous Ag^+^ with culture filtrates and is preliminary evidence for the development of silver nanoparticles (Figure 2A) and the control without silver compound under the same condition was found colorless.

#### 3.2.2 Characterization of Biosynthesized Ag-NPs

Characterizations of Ag-NPs have revealed that, as shown in Figure 2A. UV spectrum due to reduction reaction of the silver ion exhibiting polydispersed Ag-NPs, with the absorption peak at 465 nm wavelength. Blue shifting in absorption efficiency of the Ag-NPs can be due to its aggregation properties and delocalization of conduction electrons. X-ray diffraction (XRD) was used to validate the crystalline nature of Ag-NPs, which showed three distinct strongest diffraction peaks with 2θ of 32.12^°^, 29.47^°^ and 28.70^°^ corresponding to the peaks in the analysis indicating the active Ag-NPs formation with the angle index of 100, 87 and 38 respectively (Figure 2B). The characterization of silver nanoparticles by XRD studies revealed the average grain size, which is estimated out to be 20.38 nm. Therefore, from the results it can be seen that the biomolecules of *Aspergillus niger SAP2211* are involved in reduction, capping and stabilization of the synthesized Ag-NPs.

FTIR spectroscopy analysis was carried out to confirm that the culture supernatant of *A. niger* has the ability for reduction of silver in the synthesis of silver nanoparticles and to identify the potential functional groups of the biomolecules responsible for its reduction and capping. FTIR reveals multiple stretches of inverse transmission peaks at 524.95, 623.59, 725.76, 771.97, 1,030.79, 1,146.69, 1,309.16, 1,384.69, 1,572.10, 1872.18, 2,311.43, 2,741.88, 2,912.46, 3,210.55, 3,743.07, and 3,841.64 cm^−1^ which were interpreted for the identification of the functional moieties in the air-dried silver nanoparticles (Figure 2C). FTIR transmission plot shows broad intense peak at 3,210.55 cm^−1^ which indicates the presence of amine (–NH) groups of the primary amide due to proteins of *A. niger* and 2,912.46 cm^−1^ corresponds to O–H stretching of carboxylic group of protein. The reports documented that amino acid residues and peptide carbonyl groups have a strong affinity to bind with metals and serve as an encapsulating agent, thus preventing agglomeration of nanoparticles (Mitra and Das, 2008; Bozanic et al., 2010). The extracellular protein present in the fungal extract has strong ability to bind Ag-NPs either through its free amine or cysteine groups and could be responsible for the reduction of silver ions, Ag^+^ into nanosized silver particles (Jaidev and Narasimha, 2010). Stretching vibration of nitro group gives a sharp inverse peak at 1,309.16 cm^−1^ and a broad peak at 1,572.10 cm^−1^. Peak at 1,030.79 cm^−1^ can be assigned to alkoxy C–O stretching, peak at 2,741.88 cm^−1^ demonstrates O-H stretch of carboxylic acid and 2,311.43 cm^−1^ corresponds to C≡N stretching, 1872.18 cm^−1^ shows the weak C=O stretching thereby confirming the presence of aromatic compound. Inverse peaks at 1,146.69 cm^−1^ and 1,030.79 cm^−1^ were assigned to alkoxy C–O stretching (Figure 2C). Peaks at 771.97 cm^−1^, 725.76 cm^−1^ and 623.59 cm^−1^ are due to C–H bending of aromatic compound. Stretching vibrations observed at 524.95 cm^−1^ can be attributed to reduction of Ag^+^ to Ag^0^. Our results are consistent with the earlier reports for fungal-mediated synthesis of Ag-NPs.

**FIGURE 3:**
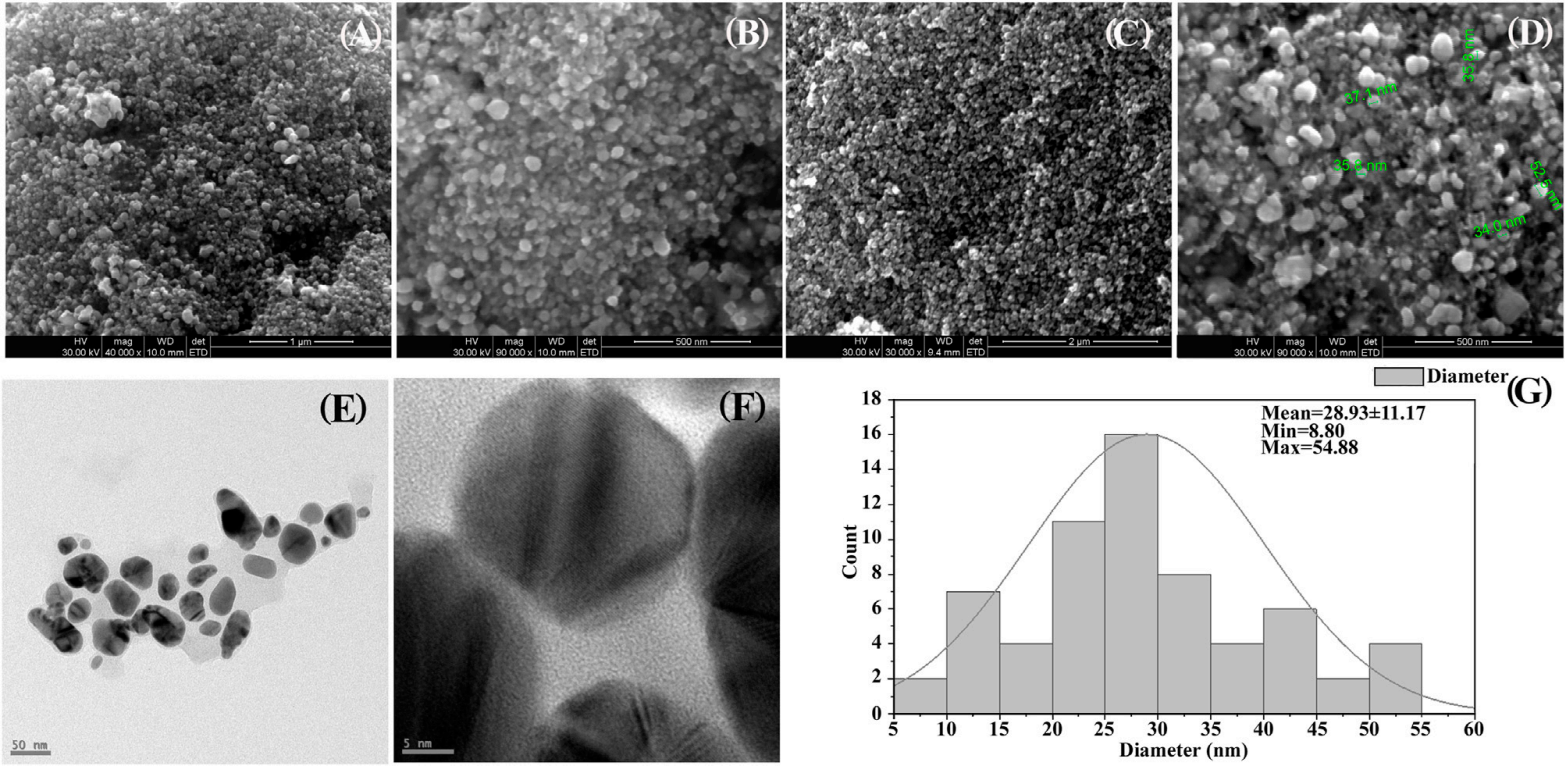
**(A–D)** SEM images showing shape and size of prepared silver nanocomposite. **(A)** 1 µM **(B, D)** 500 nm, **(C)** 2 µM. **(E,F)** TEM images **(E)** at 50 nm and **(F)** at 5nm, **(G)** Histogram particle size distribution of TEM.

SEM analysis reveals the morphological structure of the synthesized Ag-NPs nanostructure with uneven surface topology. SEM observation clearly exhibits a size range from 9.2 to 50 nm (13.53 nm ± 4.08) which are spherical and oval in shape showing aggregation ofAg- NPs (Figures 3A–D). TEM described that the average size of the well dispersed Ag-NPs was 30.31 nm ± 3.36 with different morphologies (Figures 3E,F). The size distribution was calculated using TEM images and was found to range from 8 to 55 nm (Figure 3G).

### 3.3 Assessment of Antibacterial Activity

**FIGURE 4:**
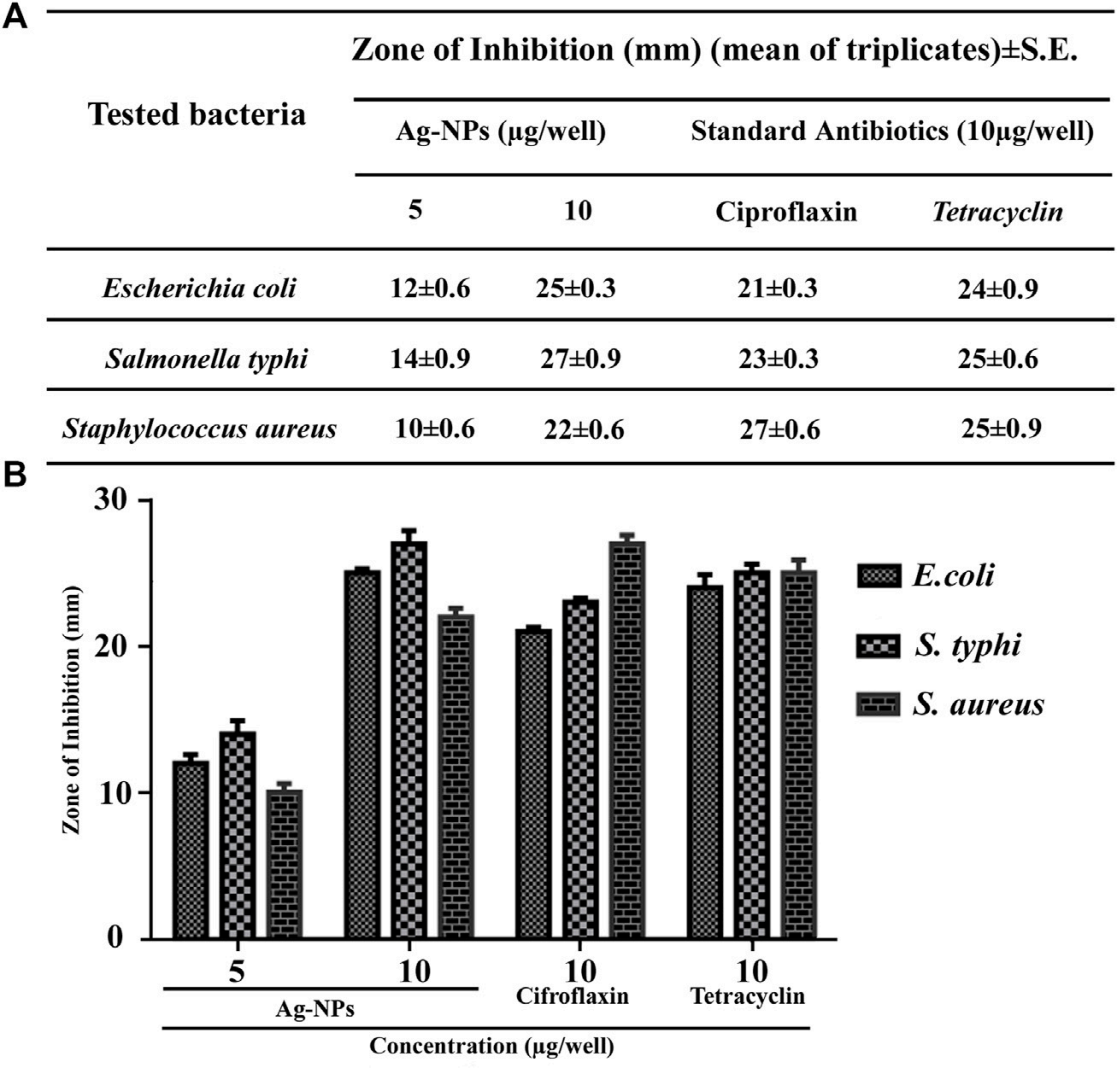
**(A,B)** Tabular and bar histogram representation showing zone of inhibition of Ag-NPs with respect to standard antibiotics against the tested pathogenic bacteria.

Antibacterial activity of prepared Ag-NPs (5 µg/well and 10 µg/well) was investigated against both gram negative (*Salmonella typhi, Escherichia coli*) and gram positive (*Staphylococcus aureus*) pathogenic bacteria by agar well diffusion method, and compared with standard antibiotics like tetracycline and ciprofloxacin (10 µg/well). The Ag-NPs were found to be effective at all concentrations for the studied bacteria (Figures 4A,B) and demonstrated strong antimicrobial potential over gram-negative bacteria by exhibiting maximum zone of inhibition for *S. typhi* at 10 µg/well compared to standard antibiotics.

### 3.4 Cytotoxicity Assessment of Ag-NPs

**FIGURE 5:**
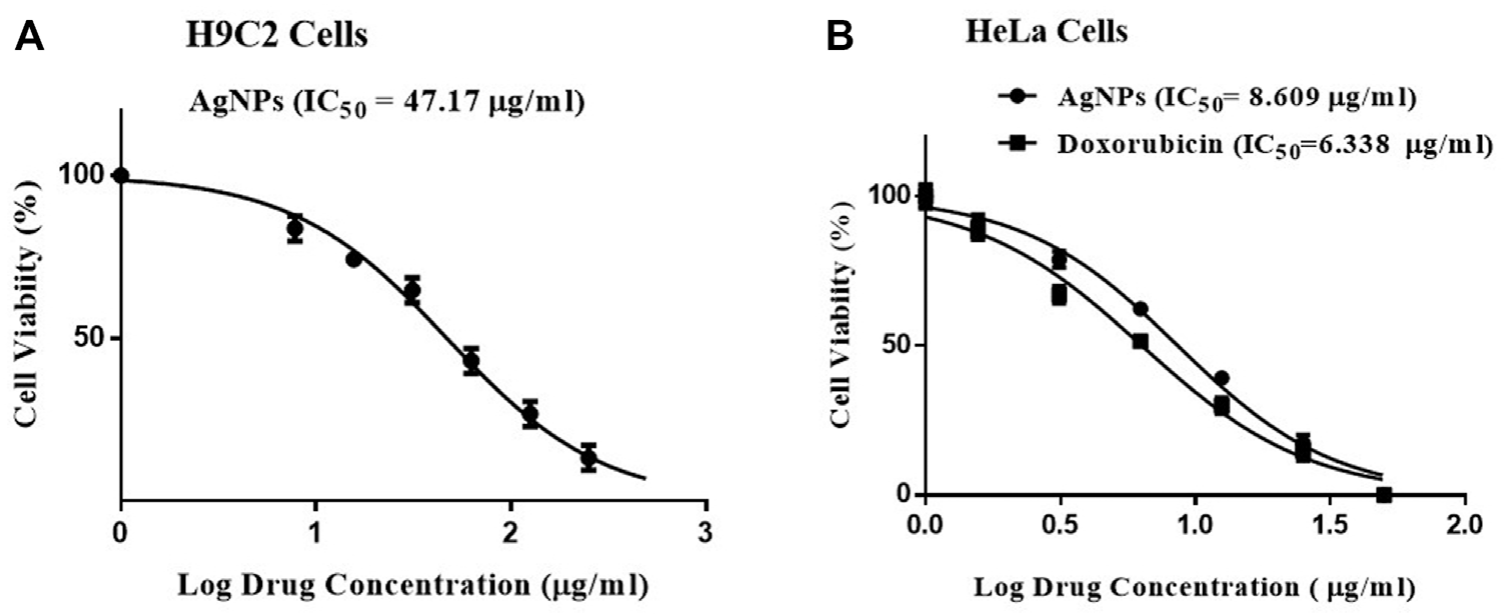
**(A,B)** Cytotoxicity measured *in vitro* by MTT assay. **(A)** Biogenic Ag-NPs on H9C2 cell lines, **(B)** Biogenic Ag-NPs and doxorubicin treatment on HeLa cell lines.

The cytotoxic effect of Ag-NPs was screened against H9C2 and HeLa cell lines by MTT assay taking doxorubicin as positive control. The Ag-NPs exhibited inhibition with IC_50_ values of 47.17 µg/ml in H9C2 (Figure 5A) and 8.609 µg/ml in HeLa cell lines (Figure 5B) respectively, and positive control showing IC_50_ of 6.338 µg/ml (Figure 5B). The results clearly exhibits that biogenic Ag nanocomposite displayed good cytotoxicity comparable to that of standard drug doxorubicin toward HeLa cell lines in dose-dependent manner after 24 h incubation. On the other hand, the effect of biogenic Ag-NPs have shown lesser effect on H9C2 cells thereby proving that Ag-NPs is non-toxic to the normal cells.

### 3.5 Apoptosis Analysis by DAPI

**FIGURE 6:**
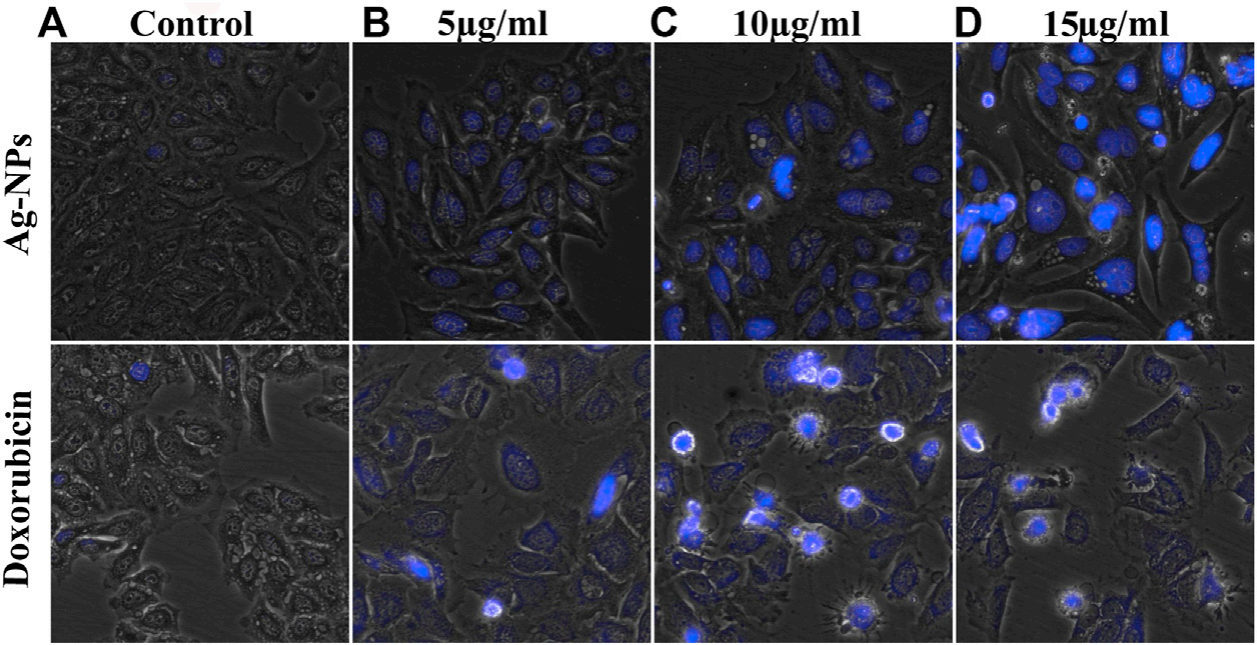
**(A–D)** Morphological changes observed in DAPI stained HeLa cells exposed to the doses of **(A)** 0, **(B)** 5, **(C)** 10 and **(D)** 15 µg/ml of both Ag-NPs (in upper row) and doxorubicin (in lower column).

DAPI staining technique was used to observe the nuclear morphological change associated with apoptosis. This dye specifically stains nuclei and binds to AT-rich region in the minor groove of DNA and its fluorescence increases 20-fold. Enhanced cell permeability of DAPI with increased apoptosis was observed thus inducing intense blue fluorescence in all Ag-NPs treated cells similar to doxorubicin treated cells (Figures 6B–D), in contrast to the effect shown on the untreated cells (Figure 6A). The DAPI staining of HeLa cell lines treated with Ag-NPs clearly indicates the induction of apoptosis by showing condensed nucleus, morphological changes and loss in cell structure (Figures 6B–D). The outcomes of DAPI staining exhibit that Ag-NPs treatment increased the count of apoptotic cells in treated cell in a dose dependent manner, in comparison to the untreated control cells and the effect shown was found to be close to the effect demonstrated by the intermediary dose of doxorubicin, thus indicating DNA damage caused by apoptosis.

### 3.6 Scratch Wound Healing Assay

**FIGURE 7:**
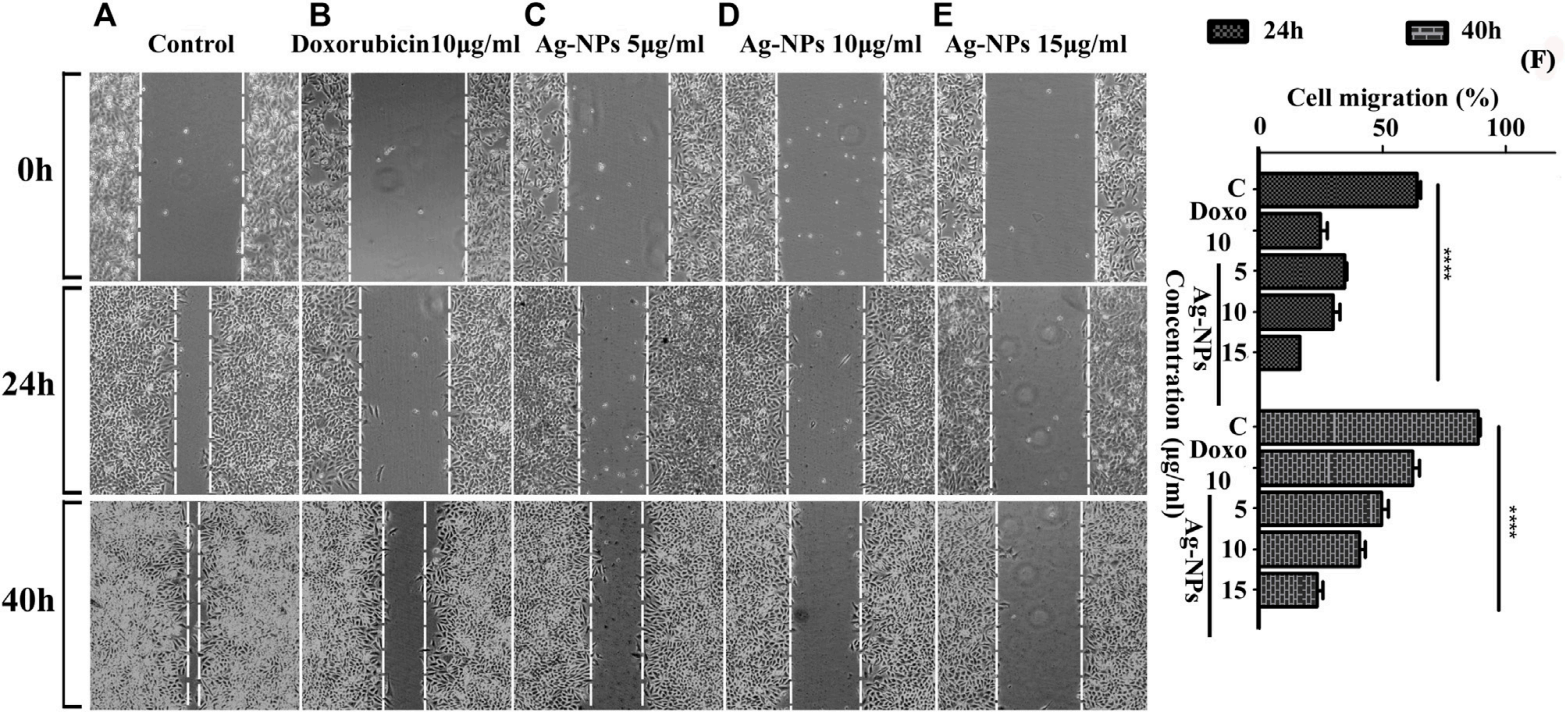
**(A–F)** Micrographs showing the measurement of scratched cell migration in the absence or presence of Ag-NPs post treatment for 24 and 40 h in HeLa cell lines. **(A)** Control at 0, 24, and 40 h, **(B)** doxorubicin 10 μg/ml, **(C)** 5 μg/ml Ag-NPs, **(D)** 10 μg/ml Ag-NPs and **(E)** 15 μg/ml Ag-NPs. **(F)** The rate of wound healing was evaluated by differences of cells filling the scratched area (*p* < 0.05) in HeLa cells in three biological replicates for each treatment and control group based on two-way ANOVA followed by Tukey’s test. Values are represented as mean % of cell migration of three replicates.

Wound healing is characterized by cell proliferation and its migration. The scratch assay has been widely used to study the *in vitro* wound closure effect. In this study, we examined the effect of Ag-NPs on HeLa cell lines migratory behavior by conducting a cell migration scratch assay. Figure 7 illustrates the microscopic images of the scratch assay and showed that Ag-NPs considerably inhibited HeLa cells migration in dose and time dependent manner after 24 and 40 h of incubations with respect to untreated control cells (Figure 7A) and doxorubicin treated cells (Figure 7B). Ag-NPs significantly reduced the cell migration at a concentration of 5, 10, and 15 µg/ml, the scratch was first measured after a period of 24 h incubation, and the migration was found to be 34.68% (Figure 7C), 29.86% (Figure 7D) and 16.10% (Figure 7E) respectively (shown in 2nd row). After another 40 h of incubation, the closure of the opened scratched area was measured out to be 49.78% (Figure 7C), 40.66% (Figure 7D) and 23.44% (Figure 7E) with respect to 5, 10, and 15 µg/ml of Ag-NPs treatments (shown in 3rd row). The positive control (doxorubicin) showed migration percentage of 24.73% (Figure 7B) at 24 h and 62.33% (Figure 7B) at 40 h, and that of negative control with 64.17% (Figure 7A) and 88.94% (Figure 7A) of migration at 24 and 40 h respectively. The comparative cell migration percentages in treated and control cells post 24 and 40 h incubations are represented in bar histogram (Figure 7F). The results suggest that synthesized Ag-NPs arrest migration of HeLa cells at doses even lesser than the IC_50_. These results clearly indicate that the synthesized Ag-NPs possess potential anti-migration property equivalent or more effective than the drug doxorubicin.

### 3.7 Transwell Matrigel Invasion Assay

**FIGURE 8:**
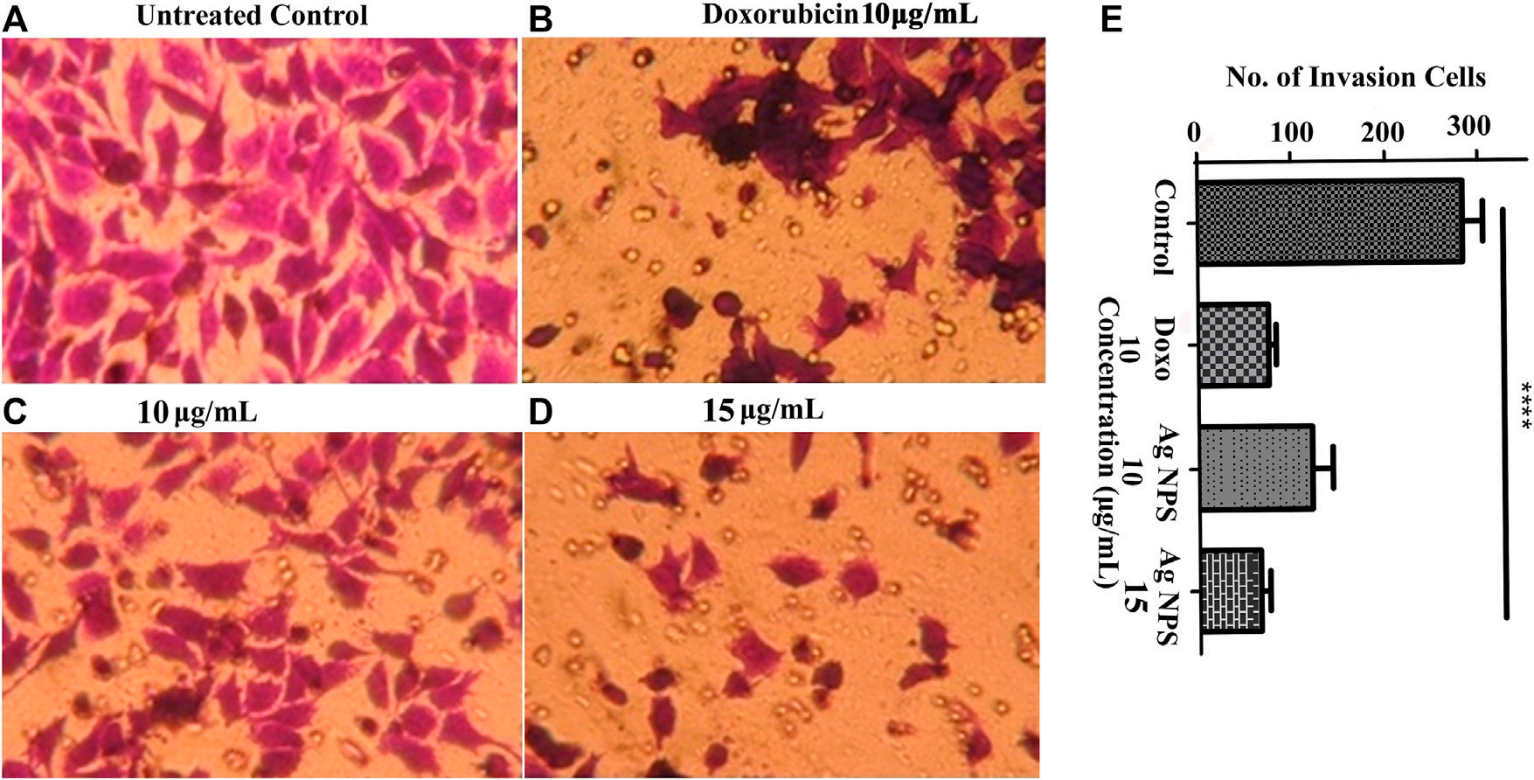
**(A–E)** Transwell assay was performed to evaluate the effect of Ag-NPs on cell invasion. **(A)** Untreated control cells **(B)** 10 µg/ml doxorubicin **(C)** 10 µg/ml Ag-NPs **(D)** 15 µg/ml Ag-NPs. **(E)** Bar histogram represents the cell count of cell invasion assay in treated and control.

Transwell invasion assay was performed to assess the Ag-NPs effect on the invasive ability of the HeLa carcinoma cells and its response to chemoattractant. The invasiveness of HeLa cells was investigated under the effect of 12% FBS used as a chemoattractant with Ag-NPs taken at 10 and 15 μg/ml concentration. The migrated cells in the membrane were stained with crystal violet and counted. In untreated control cells, the quantitative count of invasive cells in 12% FBS was found to be 282 ± 12.72 (Figure 8A). The effect of Ag-NPs 10 μg/ml with 12% FBS on HeLa cells was found to be 121 ± 11.67 and for Ag-NPs 15 μg/ml was found to be 65 ± 6.11 cells in Figures 8C,D respectively) and for doxorubicin (10 μg/ml) was found to be 74 ± 4.81 as seen in Figure 8B. Higher dose of Ag-NPs showed a significant decrease in invasion rate than the lower doses of Ag-NPs (Figure 8E). These results suggest that FBS chemoattractant increases the cell invasion capacity of the cells but this effect is attenuated with the Ag-NPs treatment, thus reducing the migration rate similar to the conventional anti-cancerous drug doxorubicin. Thus, the Ag-NPs doses significantly inhibited invasion of HeLa cells in a dose-dependent manner. Our study provides insight into the use of these green synthesized silver nanoparticles as an effective alternative treatment to suppress metastatic cervical cancer.

### 3.8 Apoptosis Assay by Annexin V FITC/PI

**FIGURE 9:**
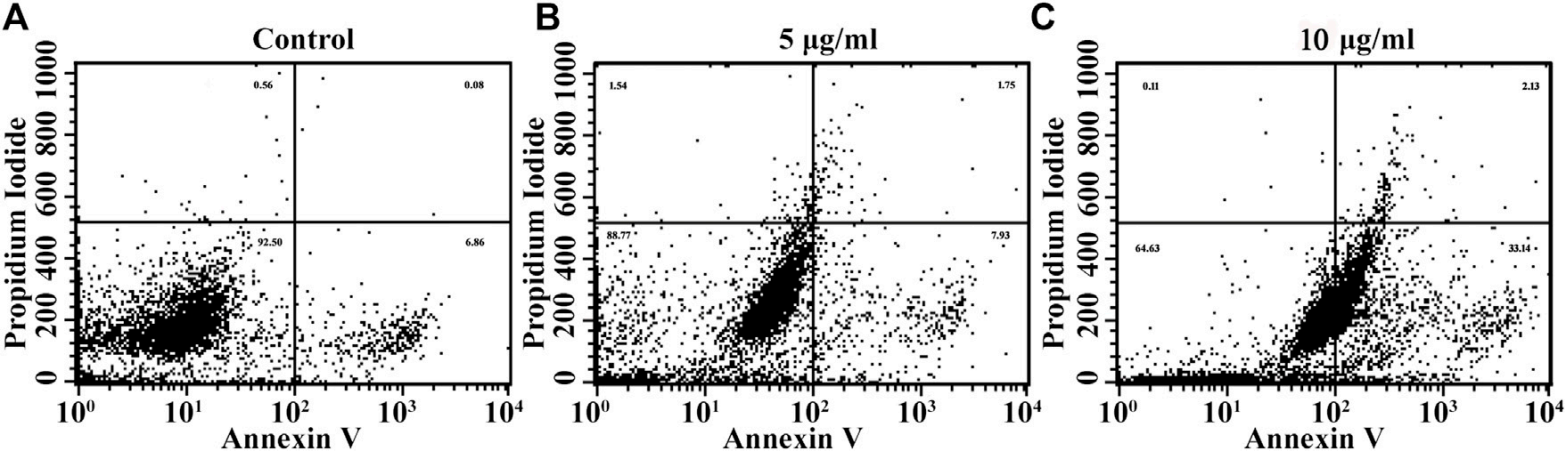
**(A–D)** Apoptotic cell death induced due to Ag-NPs treatments in HeLa cells after 24 h incubation was assessed using Annexin V FITC/PI dual staining. The percentage of early and late apoptotic cell population in the control group found to be lower, and the viable cell count was higher in control cells compared with Ag-NPs treated groups. **(A)** Control, **(B)** 5 µg/ml Ag-NPs, **(C)** 10 µg/ml Ag-NPs.

The effect of biogenic Ag-NPs on apoptosis was evaluated with Annexin V/PI double staining method. The dot blots shown in flow cytometric analysis demonstrate the different stages of apoptosis that the treated HeLa cells underwent. The lower left quadrant shows only viable cells and hence considered negative for dual stain (Annexin (−) PI (−)) and lower right demonstrates early apoptotic stage (Annexin (+) PI (−)), upper left quadrant shows necrotic cells (Annexin (−) PI (+)) and upper right (Annexin (+) PI (+)) gives count for late apoptotic phase. Results revealed that Ag-NPs treatment on HeLa cells for 24 h with 5 and 10 µg/ml Ag-NPs induced apoptosis in a concentration dependent manner (Figures 9A–C). Enhanced cell count in early stage of apoptosis was found in lower dose (5 µg/ml) i.e., 7.93% (Figure 9B) in comparison to higher dose (10 µg/ml) of Ag-NPs showing 33.14% (Figure 9C **)** of early apoptotic population in Ag-NPs treatments, with control demonstrating 6.86% (Figure 9A). It is clear from the results that the cells from the early apoptosis at higher dose have entered into the second quadrant, i.e. late apoptosis phase and were measured to be 1.75% (Figure 9B) in 5 µg/ml and 2.13% (Figure 9C) in 10 µg/ml while in positive control it is 0.08% (Figure 9A). It is evident that synthesized Ag-NPs induced dose dependent apoptotic cell death.

### 3.9 Chick Chorioallantois Membrane Assay

**FIGURE 10:**
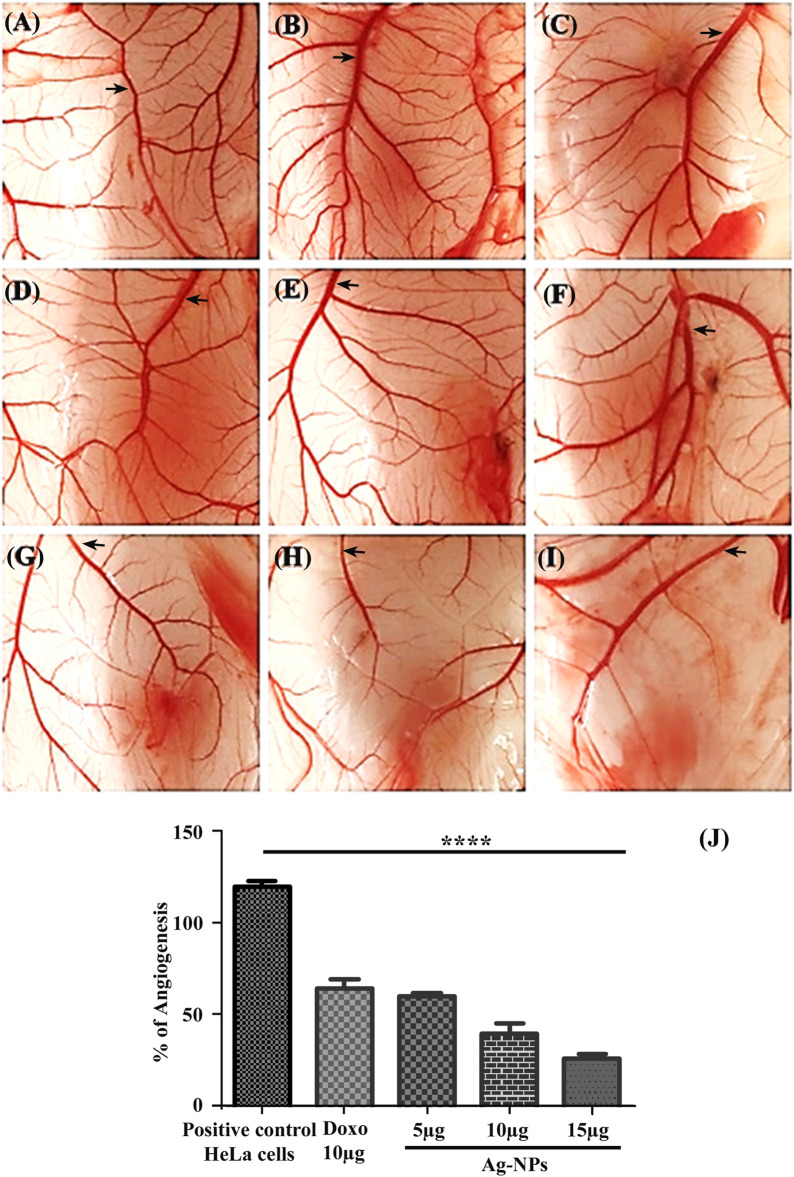
**(A–J)** Illustration of the CAM assay. **(A)** Untreated, **(B)** Treated only with HeLa cells, **(C)** Exposed to 10 µg/ml doxorubicin, CAM treated with different concentrations of Ag-NPs—**(D)** 5 µg/ml, **(E)** 10 µg/ml, **(F)** 15 µg/ml, HeLa cells inoculated CAMs treated with Ag-NPs—**(G)** 5 µg/ml, **(H)** 10 µg/ml, **(I)** 15 µg/ml. **(J)** Bar histogram showing percentage of Angiogenesis.

The CAM assay is a dependable method to study angiogenesis *in vivo* with inhibitors and stimulators. The negative control in this experiment was eggs that had not been exposed to HeLa cells, and the vascular development in the negative control was assumed to be 100% for the measurement of angiogenesis percentage. Comparison of the mean of the number of blood vessel and percentage of angiogenesis are shown in Table 1, for the negative control it is 88 ± 2.1, 100% (Figure 10A) and for positive control it is 106 ± 3.3, 119.83% (Figure 10B), the treatment groups are the eggs with induced HeLa cell for doxorubicin is 56 ± 2.8, 64% -Figure 10C) and with the Ag-NPs exposure over normal CAM cells (without HeLa cell) at a concentration of 5 µg/ml (83 ± 2.9, 95.33%-Figure 10D), 10 µg/ml (81 ± 2.9, 92.07%-Figure 10E) and 15 µg/ml (78 ± 1.3, 88.59%-Figure 10F). Results from NPs treated HeLa inoculated CAM cells illustrated significant dose dependent reduction in blood vessels formation. The Ag-NPs dose on cancer induced CAM at 5 µg/ml (Figure 10G), 10 µg/ml (Figure 10H) and 15 µg/ml (Figure 10I) showed blood vessels counts of 53 ± 2.5, 35 ± 4.9 and 23 ± 2.2 respectively with 60.34, 40.17, and 26.24% angiogenesis. Figure 10J shows comparative percent of angiogenesis in both controls and treatments. No significant changes were observed in Ag-NPs treated normal CAM, but the synthesized biogenic Ag-NPs showed a dose dependent significant decrease in vascularization (*p* < 0.05) of HeLa cells inoculated CAM cells. The CAM arrangement tends to be clustered with obtruded blood vessel formation or destroyed vascular organization in all the effective dosages of nanoparticles in comparison to control groups. According to our findings, these silver nanoparticles have a dose-dependent cytotoxic impact on blood vessel endothelial cells, limiting blood vessel development in CAM.

**TABLE 1:** Percentage of angiogenesis and reduction in blood vessel formation assessed using CAM assay.

S.No	Treatments	No. of blood vessels formation ±S.E.	% of Angiogenesis
1	Untreated negative Control	88 ± 2.1	100.0
2	Positive control (HeLa cells-1×10^6^)	106 ± 3.3	119.83
3	Doxorubicin	56 ± 2.8	64.0
4	Ag-NPs 5 µg/ml	83 ± 2.9	95.33
5	Ag-NPs 10 µg/ml	81 ± 2.9	92.07
6	Ag-NPs 15 µg/ml	78 ± 1.3	88.59
7	Ag-NPs 5 µg/ml + HeLa cells	53 ± 2.5	60.34
8	Ag-NPs 10 µg/ml + HeLa cells	35 ± 4.9	40.17
9	Ag-NPs 15 µg/ml + HeLa cells	23 ± 2.2	26.24

## 4 Discussion

Many bioactive compounds synthesized from marine species, plants and microorganisms have been investigated for their cellular and biological activities to be used as new therapeutic agents. Marine sponges are filter feeders for the substantial numbers of bacteria, fungi and suspended algae in water and share a symbiotic relationship with these microorganisms. Unexplored aquatic habitats have become an attractive source for extracting and synthesizing novel metabolites to combat antibiotic resistance and viruses with strong disease potential. Marine fungi are one of the best sources of new compounds for research (Bovio et al., 2019). *A. niger* isolated from marine sponge are well documented for natural products like asperic acid, asperazine, hexylitaconic acid, and malformin C, 3,3′-bicoumarin bicoumanigrin, 6-pyridinone derivates (aspernigrins A and B), furan and pyrano [3,2-b]pyrroles pyranonigrins (Hiort et al., 2004). These compounds of sponges represent pharmacological properties, as these compounds exhibit antibacterial, antiviral, antifungal, antimalarial, antitumor (Uchoa et al., 2017), immunosuppressive, and cardiovascular activities (Anjum et al., 2016). This study is focused on *Aspergillus strain* MK503444.1 isolated from marine sponge, and evaluation of its bactericidal activity induced by the Ag-NPs thus paving a way to overcome the grave global concern of MDR. The anti-cancerous and anti-angiogenic property of the synthesized Ag-NPs were established against cervical HeLa cell lines and were found to be considerably non-toxic in normal myocardial cell line H9C2.

The observations reveal that Ag-NPs exhibit dark reddish-brown colouration based on NPs size and concentration. Color change is due to excitation of surface plasmon resonance (SPR) of the synthesized Ag-NPs (Mulvaney, 1996). The UV–vis spectroscopy technique representing the formation and stability of Ag-NPs in suspension showed SPR peak of silver nanoparticles at 460 nm, specifying successful silver nanoparticles synthesis (Saravanan and Nanda, 2010). The size and shape of the silver nanoparticles reflect the absorbance peak with an increase in particle size, the SPR peak shifts to longer wavelengths (Kerker, 1985; Brause et al., 2002; Sosa et al., 2003). Results obtained from SEM and TEM images revealed that the synthesized Ag-NPs is of nanometer size with diameter below 100 nm, and few NPs were individually seen while majority others in aggregates form of variable size(s) and shapes (of different geometry ranging from spherical to oval shape) to ensure its stabilization by a capping agent. The crystalline nature of Ag-NPs is established by XRD demonstrating face centred cubic structure of silver based on Bragg’s reflection (Gurunathan et al., 2009a; Kalishwaralal et al., 2010). FTIR spectral data determined the associated functional groups of the biosynthesized Ag-NPs using *A. niger*. Vanaja et al. (2013) reported that the carboxylic and amine groups are the functional biomolecules found to be associated with reduction of silver ions, as determined by FTIR spectrum. Absorption peak observed via FTIR could be attributed to the reduction of Ag^+^ ions and gives chemical purity and composition constituents of Ag-NPs. The literature review confirmed the secondary metabolites are responsible for the reduction of AgNO_3_ to Ag-NPs and play a crucial role in optimizing the size, shape and stability of the biosynthesized Ag-NPs.

Antimicrobial test of green synthesized Ag-NPs at two distinct concentrations assayed by agar well diffusion methodology depends on the particle size of the NPs. NPs with small particle size and large surface area can easily enter the cell and produce more effective bactericidal activity than the larger particles (Toshikazu, 1999). Results reveal that the synthesized Ag-NPs can be used as an effective antimicrobial agent. The zone of inhibition was found to be more at higher concentration of Ag-NPs. Data also revealed that these Ag-NPs are more toxic towards gram-negative bacteria in comparison to gram-positive, which might be attributed to differences in cell wall composition. Gram-negative bacteria contain thin layer of peptidoglycan facilitating the Ag^+^ ions to enter the cell and lipopolysaccharide layer comprising of negative charge which attracts Ag^+^ ions causing enhanced uptake leading to destruction of cell wall (Sosa et al., 2003; Vanaja et al., 2013) The mechanisms of NPs uptake by pathogenic bacteria caused cell death, inhibition of cell cycle (Kumbhakar et al., 2017), induced oxidative stress, induced proton leakage (Dibrov et al., 2002) and reactive oxygen species (ROS) formation (Gurunathan, 2019) in cellular defense. Reports described that Ag-NPs adhere to the cell surface via electrostatic attraction and produces ROS (superoxide and hydroxyl radicals, hydrogen peroxide) that interrupt the cell permeability and respiration, thus displaying robust bactericidal activity, and also destroys the biofilm formation (Kalishwaralal et al., 2010). It can damage the nucleic acids by binding with the thiol group of cysteine, thus resulting in impairment of protein synthesis (Feng et al., 2000; Kulthong et al., 2010).

Park et al. (2010) documented that Ag-NPs (also known as “Trojan-horse”) trigger cancer cells following intracellular uptake of Ag-NPs and subsequent release of its reactive Ag^+^ ions resulting in ROS production and ultimately leading to apoptosis. *In vitro* cytotoxicity of the tested Ag-NPs showed dose dependent cytotoxicity in HeLa cells without much effect on normal cells. Few *in vitro* studies have reported that Ag-NPs synthesized using marine sediment have significant toxicity against HepG2 cell lines upto 85% (Anand et al., 2015) with IC_50_ of 500 mg/ml and in lymphoma ascites tumor model with IC_50_ of 300 mg/ml (Sriram et al., 2010). Previously, Ag-NPs synthesized from *S. cumini* lead to destruction of cellular components and generation of reactive oxygen species. DNA damage is an indicator of apoptosis induction and is confirmed by DAPI staining. Scratch assay results establish the significant anti-migratory property of the Ag-NPs in both time and dose dependent manner on the HeLa cell lines. Our data corroborates with the previous published report of the biosynthesized Ag-NPs showing a dose dependent cytotoxicity due to enhanced ROS and oxidative DNA damage in BEAS-2B cells (Kim et al., 2011) and human keratinocytes (Sapkota et al., 2017), leading to apoptotic mediated cell death (Jacob et al., 2012). The integrity of plasma membrane is determined via annexin V conjugated with PI and hence used for the detection of apoptotic cell population. Data from the study revealed that the biogenic Ag-NPs were found to induce apoptosis in the HeLa cells in dose dependent manner, which is in accordance with other previous reports (Çiftçi et al., 2013). Apoptosis induced due to Ag-NPs treatments leads to the release of phosphatidylserine (PS) from the inner cytoplasmic membrane in treated cells and the externalized PS binds specifically to Annexin-V conjugated with propidium iodide; whereas in untreated control cells the PS residue are present inside the inner membrane of cytoplasmic membrane and hence not detected as apoptotic cells (Baharara et al., 2018). Necrotic cells are generally leaky hence PI binds DNA but as PS does not flip out hence is not stained by Annexin V.

One of the most important challenges in cancer therapy is to prevent invasion and angiogenesis of cancer cells. Our data show potential inhibitory effects of Ag-NPs against both invasion and angiogenesis. In previous studies it has been reported that in case of normal non-cancerous cells, vascular endothelial growth factor (VEGF) usually binds to the receptor of endothelial cells by activating the PI3K/Akt pathway and thereby lead to angiogenesis (Asharani et al., 2009; Gurunathan et al., 2009b). Gurunathan et al., 2009b stated that Ag-NPs eventually interrupt the angiogenesis and retards the oxygen supply to tumor cells, ultimately resulting in tumor cell death. Ag-NPs have been documented to show genome instability by DNA damage and chromosomal aberration, apoptosis induction due to imbalance in homeostasis, cytoskeletal instability inhibits cell cycle culminating in anti-proliferative activity of cancer cells (Asharani et al., 2009). In present investigation, we aimed and concluded that biogenic Ag-NPs do have the capacity to interrupt angiogenesis process and prohibit the invasive and metastatic potentiality of the proliferating HeLa cell specifically; further studies in other cancer cell lines are warranted to explore the understanding of interaction for safe usage of the biosynthesized Ag-NPs as a therapeutic agent.

## 5 Conclusion

The present investigation concerns with novel biosynthesized Ag-NPs using *A. niger* inhabiting marine sponge. The biogenic Ag-NPs was highly stable with size lesser than 50 nm and crystalline nature. In the biological use of Ag-NPs, we discovered that the Ag-NPs exhibits promising antimicrobial efficacy against the studied pathogenic bacteria. Meanwhile, *in vitro* and *in vivo* assessment have demonstrated concentration dependent anti-proliferative, anti-invasive, pro-apoptotic and anti-angiogenic activities against HeLa cell lines. The results provides an insight on the use of biogenic Ag-NPs for future therapeutic application, as an alternative to commercial available drugs, with which further studies needs to be carried out.

## Data Availability

Fungal rDNA-ITS sequence is available at GenBank, NCBI with accession number MK503444.1 (https://www.ncbi.nlm.nih.gov/nuccore/MK503444.1).
